# Current advances in the treatment of Alzheimer's disease: focused on considerations targeting Aβ and tau

**DOI:** 10.1186/2047-9158-1-21

**Published:** 2012-10-30

**Authors:** Yang Hong-Qi, Sun Zhi-Kun, Chen Sheng-Di

**Affiliations:** 1 Department of Neurology, Henan Provincial People's Hospital, Zhengzhou , Henan Province, 450003, People's Republic of China; 2 Department of Neurology and Institute of Neurology, Ruijin Hospital, Shanghai Jiao Tong University School of Medicine, Shanghai, 200025, People's Republic of China

**Keywords:** Alzheimer's disease, Amyloid precursor protein, Beta amyloid, Tau, Pathogenesis, Treatment

## Abstract

Alzheimer’s disease (AD) is a neurodegenerative disorder that impairs mainly the memory and cognitive function in elderly. Extracellular beta amyloid deposition and intracellular tau hyperphosphorylation are the two pathological events that are thought to cause neuronal dysfunction in AD. Since the detailed mechanisms that underlie the pathogenesis of AD are still not clear, the current treatments are those drugs that can alleviate the symptoms of AD patients. Recent studies have indicated that these symptom-reliving drugs also have the ability of regulating amyloid precursor protein processing and tau phosphorylation. Thus the pharmacological mechanism of these drugs may be too simply-evaluated. This review summarizes the current status of AD therapy and some potential preclinical considerations that target beta amyloid and tau protein are also discussed.

## Introduction

Alzheimer’s disease (AD) is a progressive neurodegenerative disease characterized clinically by insidious onset of memory and cognition impairment, emergence of psychiatric symptoms and behavioral disorder, and impairment of activities of daily living. It is the most frequent form of dementia found in the elderly. It is estimated that the prevalence of AD over the age of 85 may be as high as 25~50%, and AD is increasingly being recognized as one of the most important medical problems in the elderly. With the increasing number of elderly and growing of life expansion, more people will be suffered from AD, thus give a great economic burden to the families, the care-givers and the whole society. Since detailed molecular mechanisms underline the pathophysiology of AD are still remained to be clarified, currently available drug therapies for AD consist primarily of cholinesterase inhibitors (donepezil, galantamine, rivastigmine, Huperzine A) and an N-methyl-D-aspartate receptor antagonist (memantine) approved by the U.S. Food and Drug Administration (FDA) and some neuroprotective agents. Although these drugs did alleviate some of the psychological and behavioral symptoms of AD patients, effective pharmacological interventions for prevention and treatment of AD, that is, the disease-modifying therapies, are lacking.

During the past decade, many hypotheses have been put forward for AD pathogenesis. Among them, the β-amyloid (Aβ) cascade and the tau hyperphosphorylation are the theories that have widely been accepted. Thus the disease-modifying therapies focus mainly on the agents that will decrease Aβ content and tau hyperphosphorylation. Here we review the potential disease-modifying therapies and some compounds that are currently undergoing preclinical and clinical evaluations.

## Cholinesterase inhibitors

Post-mortem studies indicated the reduced choline uptake, acetylcholine (ACh) release and progressive loss of cholinergic neurons in AD brain. This “cholinergic deficient hypothesis” claims that the symptoms of AD is caused by decreased acetylcholine in the presynapse, thus increasing acetylcholine by inhibiting its degradation should improve the memory and cognition impairment in AD. Since 1993, the FDA approved four cholinesterase inhibitors for treatment of mild to moderate AD, the tacrine (1993), donepezil (1996), rivastigmine (2000) and galantamine (2001). But tacrine was later abandoned because of its hepatotoxicity, gastrointestinal adverse reactions, poor oral bioavailability and frequent dosing requirements. Multi-center randomized double-blind clinical trails indicated that donepezil and galantamine are effective in improving and maintaining cognitive and global function and activities of daily life as compared with control group. And the side effects with donepezil and galantamine are usually mild and taper with continued use and can be minimized by taking with food. Butylcholinesterase has also been found to degrade ACh in healthy and AD-affected brains and is an additional target for the treatment of AD. Rivastigmine is an inhibitor of both AChE and butylcholinesterase, and clinical trails showed that it was effective in improving cognition and functional impairment without apparent side effects in AD patients compared with placebo. Besides the above mentioned effects, donepezil and rivastigmine can also affect amyloid precursor protein processing, which means that the “cholinergic deficient hypothesis” may be too simple and that still other mechanisms may exit besides restoring Ach balance. The cholinergic system may play a role in the production of two proteins responsible for neurotoxicity in AD: Aβ and tau. Except the drugs approved by the FDA, still many other cholinesterase inhibitors are under clinical trials. For example, phenserine treatments increased cognition and regional cerebral metabolic rate for glucose in AD patients. Clinical trial with Dimebon, a cholinesterase inhibitor and also a NMDA-antagonist, showed improved cognitive and self-service functions while diminishing the psychopathic symptoms in AD patients. Huperzine A, a Chinese herb with reversibly and selectively acetylcholinesterase inhibition activity, displayed good pharmacokinetics with a rapid absorption and a wide distribution in the body at a low to moderate rate of elimination. Clinical trials have shown its cognitive enhancement in AD at a dose of 0.4 mg and seems to be a potential treatment option for AD. The development of the multimodal drug, ladostigil, combined neuroprotective effects with monoamine oxidase (MAO) -A and -B and cholinesterase inhibitory activities in a single molecule, was tested in Phase II clinical trial intended for AD, and the results are unpublished yet. Donepezil was first developed for mild to moderate (5~10 mg/d) AD patients, but larger dose (23 mg/d) was recently approved in the U.S. for treatment of moderate to severe AD. The good safety and predictable tolerability profile in a phase 3 trial for donepezil 23 mg/d supports its favorable risk/benefit ratio in patients with advanced AD [1]. Rivastigmine is effective in improving the cognitive and global functioning in AD patients, but the incidence of nausea and vomiting make it difficult to maintain high therapeutic doses in clinical practice. By delivering the drug through the skin and directly into the bloodstream, transdermal patches resulted in reduced rates of nausea and vomiting compared with oral ChEIs and represents a next generation of acetylcholinesterase treatments. In a four week prospective, multicenter, observational study on transdermal rivastigmine in Germany, 11.7% of patients had adverse evens, mainly affecting the skin or the gastrointestinal tract. Only a minor proportion of patients discontinued therapy due to adverse evens. With rivastigmine treatment, the percentage of patients taking psychotropic comedication decreased and these results were in line with data from controlled clinical trials. Still other candidates are being tested and the data are to be published.

## Aβ-targeting strategies

**Figure 1:**
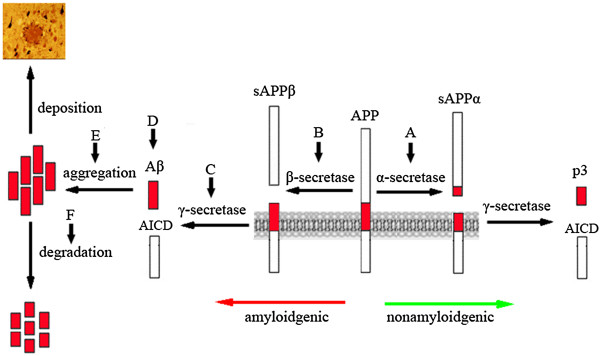
**The Aβ-targeting strategies based on the Aβ cascade hypothesis.** The processing of APP and the production, degradation of Aβ are illustrated and the therapeutic potential of targets are indicated with vertical arrows marked by alphabet: **A**, α-secretase activators; **B**, β-secretase inhibitors; **C**, γ-secretase inhibitors/modulators; **D**, immunotherapy; **E**, Aβ-aggregation inhibitors; F, Aβ-degradation activators. Modified and reproduced with permission from Yang et al. (2012) and see reference [2] for further details.

One of the hallmarks of AD is the presence of senile plaques in the hippocampus, which are primarily formed from the extracellular deposition of Aβ, a 40-42(3) amino acid polypeptide. The Aβ is deprived from a large transmembrane protein, the amyloid precursor protein (APP) by sequential proteolysis of two proteases, the β- and γ-secretase, at the N- and C-terminus of the Aβ sequence respectively. Alternatively, APP can also be processed by α-secretase within the Aβ sequence and thus not only preclude the formation of Aβ peptide but also generate a soluble neurotrophic sAPPα. Many experiments indicated that Aβ is a neurotoxin; it aggregates and forms deposits that finally lead to neuronal dysfunction. The pathological accumulation of Aβ in the brain leads to oxidative stress, neuronal destruction and finally the clinical symptoms of AD. Following this hypothesis, secondary prevention of AD can be made by: decreasing the production of Aβ, stimulation of clearance of Aβ formed or prevention of aggregation of Aβ into amyloid plaques (Figure 1). The corresponding targets are also indicated in the figure.

### β-secretase inhibitors

The therapeutic potential of β-secretase (also named β-site APP cleaving enzyme, BACE1) inhibitor has been suggested by several studies. BACE-knockout mice develop normally, showed no consistent phenotypic differences from their wild-type littermates and produced much less Aβ from APP. Lateral ventricles injection of BACE inhibitor led to a significant dose- and time-dependent lowering of brain Aβ40 and Aβ42, a robust decreased sAPPβ and an increased sAPPα secretion. While injection of another inhibitor KMI-429 into the hippocampus of APP transgenic mice reduced Aβ production. Although the data were positive both in animal and cells levels, the drugs used are mostly small peptides and are not appropriate for clinical application with their ability of penetrating the blood–brain barrier are susceptible. Development of new drugs especially small molecules which can penetrate the blood–brain barrier is wanted. Recently in APP transgenic mice, oral administration of non-peptidic BACE1 inhibitor, GSK188909, results in a significant reduction in the level of Aβ40 and Aβ42 in the brain. PMS777, a new cholinesterase inhibitor with anti-PAF activity, could decrease sAPPα secretion and Aβ42 release in SH-SY5Y^APP695^ cells and PC12 cells although its effect on BACE1 is not clear [3]. This neuroprotection in increasing the memory and cognitive ability in demented mice induced by scopolamine as evidenced by Morris water-maze test suggested its therapeutic potential. Inhibition of APP dimerization by small molecules resulted in reduction in Aβ levels as measured by ELISA. This effect is accompanied by lowered sAPPβ levels, suggesting that blocking the dimerization can prevent the cleavage by β-secretase in the amyloidogenic processing of APP. Thus APP dimerization inhibition may be a viable means of reducing Aβ production [4]. Recently the mechanisms of tight regulation of BACE1 from transcriptional, post-transcriptional and post-translational levels are increasingly illustrated and may provide new insights for clinical application in the near future [5].

### γ-secretase inhibitors/modulators

Treatment of AD mice/rats with γ-secretase inhibitors DAPT resulted in decreased Aβ levels in plasma and cerebrospinal fluid (CSF). The data is also positive in other γ-secretase inhibitors like BMS-299897 and MRK-560. LY450139 dihydrate, another γ-secretase inhibitor, was studied in a randomized, controlled trial of 70 patients with mild to moderate Alzheimer disease. And the results indicated that it decreased 38% plasma Aβ40 and 4.5% CSF Aβ40, and the treatment was well tolerated. Recruitment of double blind placebo controlled Phase III study of tarenflurbil in patients with mild AD showed no benefit on cognitive or functional outcomes and the program was thus discontinued following this disappointing. Notch, which is necessary for growth and development, is also a substrate of γ-secretase. Notch related side effects of γ-secretase inhibition (e.g. severe gastrointestinal and haemopoetic side effects, neurodegeneration) have been hampering the development of clinically useful γ-secretase inhibitors so far. Thus the drug development is now focusing on the development of γ-secretase modulators, with the purpose of shifting the γ-secretase cutting point to produce shorter, non-toxic Aβ fragments. Some non-steroidal anti-inflammatory drugs (NSAIDs) such as tarenflurbil, is a promising candidate. A phase II trial with tarenflurbil with mild to moderate AD showed a significant improvement in primary outcome of global functioning and activities of daily living, compared to placebo and a positive trend towards improved cognition. Although it reduced amyloid-β deposition in the PDAPP transgenic mouse after 5 months of administration and have a statistically significant reduction in newly synthesized Aβ in human volunteers, the results in Phase III trials demonstrated that patients who were treated with it displayed an increased deterioration in cognition and activities of daily living compared to placebo-treated controls and it was discontinued. With its very complex mode of action, it can’t be excluded that this compound-mediated worsening of the symptom is related with its effect on Notch signaling [6].

### α-secretase activators/modulators

Since α-secretase and β-secretase compete for the same substrate of APP, upregulation of α-secretase activity may decrease the amount of APP available for β-secretase, and thus decrease Aβ secretion and have therapeutic potential. Many studies had indicated that members of the adamalysin family of proteins, mainly ADAM 10, ADAM 17 and ADAM 9, fulfill some of the criteria required of α-secretase. Overexpression of ADAM10 in transgenic mice showed not only less amyloid deposition in the hippocampus and lower Aβ levels in brain homogenate when compared with APP monotransgenic mice, but also improved neurological function as evaluated by long term potentiation (LTP) and the Morris water-maze test [7]. Many studies indicated that the currently effective drug for AD all can increase α-secretase activities [8]. Our previous study showed that protein kinase C (PKC) activator TPPB could increase α-secretase activity and decrease Aβ secretion [9]. SIRT1 could activate the gene code for α-secretase ADAM10 and then suppress Aβ production in AD transgenic mice [10]. Deprenyl, a neuroprotective agent used to slow AD progress, was shown to increase α-secretase activity by promoting ADAM10 and PKCα/ε translocation [11]. As epigenetics involvement in AD pathogenesis is increasingly illustrated [12], compounds with PKC activation and HDAC inhibition properties may offer a new approach to therapies that exhibit disease-modifying effects in the treatment of AD, as opposed to symptomatic relief only [13]. This implies that stimulating α-secretase through several pathways may have therapeutic potential and the work is going on, but no clinical data available at present.

### M1 muscarinic agonists

M1 muscarinic receptors play a role in an apparent linkage of three major hallmarks of AD: Aβ peptide; tau hyperphosphorylation and loss of cholinergic function conductive to cognitive impairments. It was discussed here because it can also regulate secretase activities. Activation of M1 mAChRs with these agonists leads to enhanced secretion of sAPPα, (via α-secretase activation), to decreased Aβ (via γ-secretase inhibition), and the inhibition of Aβ- and/or oxidative stress-induced cell death. Talsaclidine is a functionally selective muscarinic M1 agonist that stimulates non-amyloidogenic α-secretase processing in vitro. In a double-blind, placebo-controlled, and randomized clinical study in AD patients, treatment with talsaclidine decreased CSF Aβ about 20% as compared with the baseline, suggesting its therapeutic potential. AF102B, another M1 agonist, also decreased CSF Aβ of AD patients, and clinical trials will determine if AF267B may become an important therapy in AD. In vitro studies indicated that these M1 agonists may act through decreasing γ-secretase and increasing α-secretase activities that finally decreased Aβ secretion. And it also decreased tau-phosphorylation.

### Aβ-aggregation inhibitors

The neurotoxic effect of Aβ has been documented on numerous occasions and thus decreasing its neurotoxicity or inhibiting its aggregation may have therapeutic potentials. The first drug was a β-sheet breaker iAβ5p, which showed that intrahippocampal injection of it resulted in improved spatial memory and decreased amyloid plaque deposits. Tramiprosate (3APS, Alzhemed) is a compound that binds to soluble Aβ and inhibits the formation of neurotoxic aggregates that lead to amyloid plaque deposition in the brain. Phase II clinical trial with mild to moderate AD patients showed that it dose-dependently reduced CSF Aβ42 levels after 3 months of treatment, but had no significant effect on CSF40, tau and psychometric scores between groups. Disappointing results of the US phase III trial in the year 2007 have led to discontinuation of the European phase III trial. Thus, the trials of other antifibrilization agents are planned.

### Immunotherapy

Both active (vaccination) and passive (monoclonal antibodies) immunization are studied in AD patients after promising data from in vitro experiments and animal studies. Active immunization against Aβ42 in transgenic mice resulted in decreased plaques and improved cognitive function as tested by Morris water maze trials. These exciting results led to clinical vaccination trials in patients with AD. A randomized, double-blind, placebo-controlled, phase II clinical trial with the synthetic Aβ peptide AN1792/QS-21 in patients with mild to moderate AD was initiated, but the trial was later discontinued because of approximately 6% of the immunized AD patients (18/300) developed meningoencephalitis [14]. And the study also indicated a dissociation between brain volume loss and cognitive function in AN1792/QS-21 antibody responders as measured by MRI scanning. Patients treated with AN1792/QS-21 showed decreased plaque pathology and only patients with meningoencephalitis showed increased T-cell activation. It was thus speculated that T-cell response may be necessary for development of meningoencephalitis. A recent completed clinical trial with Aβ42 immunization indicated that although immunization with Aβ(42) resulted in clearance of amyloid plaques in patients with Alzheimer's disease, this clearance did not prevent progressive neurodegeneration [15]. Alternatively, new modified vaccines which contains the antibody epitope(s) but lacks the T-cell reactive sites of full-length Aβ1-42 are warranted. To achieve this, a novel peptide-carrier protein using an amino-terminal fragment of Aβ has been developed to avoid potentially harmful T-cell responses, while maintaining a similar antibody response to that of AN1792. The initial data are promising and the trials are going on. Passive immunotherapy in AD patients with repeated intravenous administration of human immunoglobulin against Aβ peptide resulted in stopped cognitive decline and slight improvement in functional scores and phase I and II clinical trials with LY2062430 are also being investigated. A phase II, multicenter, randomized, double-blind, placebo-controlled clinical trials of bapineuzumab decreased total and phosphorylated tau levels in CSF without affecting Aβ level with some patients developed transient cerebral vasogenic edema [16,17]. To date, although most of immunotherapy showed cognitive function improvement and beta amyloid load reduction by PET study, the adverse events are still the question to be solved.

### Aβ-degrading enzymes

Recent studies have indicated that Aβ peptide could be degraded by a kind of protease called Aβ degrading enzyme, rather than being cleared from the vascular system by the so-called “vascular pathway”. There is a kinetic equilibrium between Aβ production, degradation and transportation within the brain and transport out of the brain. The following proteinases have the abilities of degradating Aβ peptide: neprilysin (NEP), insulin-degrading enzyme (IDE), plasmin, endothelin converting enzyme (ECE) 1 and 2 and angiotensin-converting enzyme (ACE). All these proteinase can degrade the Aβ peptide at different amino acid residues within the Aβ sequence. NEP inhibitor injection and/or NEP knockout mice showed decreased Aβ degradation and cognitive activities declining, while overexpression of NEP resulted in improved spatial memory and decreased Aβ levels. Researchers have observed decreased NEP activities with increasing age and thus besides decreasing Aβ production, increasing Aβ degradation may also have therapeutic potential. Gene transfer studies have generated positive data in transgenic mice and neuroscientist are seeking for small molecules that can stimulate NEP activities. Studies have showed that APP intracellular domain (AICD) could upregulate NEP transcription and increase Aβ degradation [18]. Imatinib, a tyrosine kinase inhibitor, was shown to elevate AICD in H4 human neuroglioma cells, and this was accompanied by concomitant increases of NEP protein, mRNA levels, and activity [19]. Valproic acid, a widely used drug in the treatment of epilepsy, was capable of up-regulating NEP expression and activity in human neuroblastoma SH-SY5Y cell lines. Injections of valproic acid to rats resulted in upregulation of expression and activity of NEP in hippocampus. Administration of valproic acid has also restored NEP activity and memory deficit in adult rats caused by prenatal hypoxia [20]. Valproic acid is a histone deacetylase (HDAC) inhibitor. Correction of AD mice behavior deficit by valproic acid implies involvement of epigenetics in AD pathogenesis. The clinical data of the effects of valproic acid and other HDAC inhibitors on AD patients are still needed. Estrogen and green tea all could increase NEP activity and suggest their potential in AD treatment but there is a long way before their final clinical application. In vitro study of neuroectodermally converted mesenchymal stem cells reported significant upregulation of NEP activity, while clinical translation of stem cell therapy still faces many obstacles as reported.

### Apolipoprotein E (ApoE) promotes Aβ clearance

Recent studies have indicated that it was the decreased clearance/degradation rather than increased production of Aβ account for its deposition in sporadic AD [21,22]. Possession of apolipoprotein E (ApoE) 4 allele increased the risk of late onset sporadic AD, but the detailed mechanisms are still unknown. Through nuclear receptor stimulation, ApoE lipidation is increased. The lipidated ApoE activates microglia and/or astrocyte to degrade Aβ. It decreased brain amyloid plaque burden and improved behavior functions in AD transgenic mice [23,24]. In addition, the effect of ApoE on Aβ clearance is also ApoE isoform-dependent: with ApoE2 the strongest effect and ApoE4 was significantly less effective in promoting Aβ clearance. The nuclear receptor-mediated, ApoE-directed therapeutics thus can decrease brain Aβ level and have disease-modifying potentials in AD prevention [24]. Bexarotene is a nuclear receptor modulator and ApoE activator, whether it is effective in AD prevention needs to be explored clinically [25,26].

### Drugs influencing Aβ blood–brain barrier transport

The receptor for advanced glycation end products (RAGE) resides in the blood vessel wall cells and transport Aβ across the blood brain barrier from systemic circulation to facilitate their accumulation in brain. In contrast to RAGE, low-density lipoprotein receptor-related protein-1 (LRP-1) mediates transport of Aβ peptide out of brain. In AD patients the RAGE is elevated while the LRP-1 is lowered. Inhibition of RAGE-ligand interaction suppresses accumulation of Aβ in brain parenchyma in a mouse transgenic model. Thus inhibition of RAGE and/or activation of LRP-1 may be a therapeutic target for AD, but there are no clinical data available at present.

### Drugs development based on the metals hypothesis

There is increasing evidence that metal (mainly Cu, Zn and Fe) metabolism is involved in the major phthophysiological events of AD: APP processing and tau hyperphosphorylation. Several chelators of Zn/Cu have been shown to inhibit Aβ aggregation in vitro and in vivo. A phase II clinical trial with clioquinol, a metal-protein-attenuating compound that inhibits zinc and copper ions from binding to Aβ, led to improved cognitive function, decreased plasma Aβ42 level and zinc concentration as compared with control group. Other metal chelators including XH1, DP-109, PBT2 and so on showed improved cognitive function and decreased CSF Aβ42 compared with placebo, but not plasma Aβ. PBT2 was an orally bioavailable, second generation 8-OH quinoline derivative of clioquinol, and is advancing as a disease-modifying candidate drug for Alzheimer’s disease.

## HMG-CoA reductase inhibitors (the “statins”)

Epidemiologic studies indicated reduced AD prevalence in individuals who take statins. In vitro studies showed that cholesterol-rich diet increased β-secretase processing of APP while cholesterol lowering resulted in decreased Aβ production. The view that increased levels of cholesterol facilitate Aβ production while statins treatment lowers Aβ production led to the hypothesis that statins may be a promising treatment for AD. Clinical trial with atorvastatin for 1 year provides some clinical benefit in AD patients. Treatment with lovastatin resulted in decreased plasma Aβ level. But still other studies did not observe the decreased plasma Aβ levels. It is difficult to explain the inconsistency between these studies; it may be related with the different cognitive test used, treatment period, experiment design, the discrepancy between studies, and also, different stages of the disease respond to statins differently. Another important factor has to be addressed here is that the dosage of statins used in clinical trial may be far too less than that used in *in vitro* culture and consequently, more different results in human trials than in *in vitro* experiment. Although the effect of statins on Aβ production is uncertain, the connection between cholesterol and AD is still supported by many studies. Thus, there is a need for larger clinical studies where both cholesterol metabolism and APP metabolism are followed during and after treatment.

## Monoamine oxidase inhibitors

MAO inhibitor deprenyl is an anti-Parkinson drug used to inhibit dopamine degradation in the brain. Also as a neuroprotective agent, deprenyl has been used to slow the progress of neurodegenerative diseases such as AD for many years. Although controversial, some clinical trials have indicated that deprenyl could alleviate some symptoms of AD. The in vitro experiment that deprenyl can regulate APP processing through PKC and mitogen activated protein kinase (MAPK) signaling pathways may explain its clinical effect in the late stage of the disease. Another MAO-B inhibitor rasagiline is a bifunctional molecule which also has acetylcholinesterase inhibition activity. Concerning its neuroprotective mechanisms, including regulation of APP processing, activation of PKC and MAPK signaling pathways, inhibition of cell death markers and upregulation of neurotrophic factors rationalize its application to AD treatment, but the clinical trial data is unpublished yet. Ladostigil is a dual acetylcholine-butyrylcholineesterase and brain selective MAO-A and -B inhibitor in vivo which was shown to antagonize scopolamine-induced impairment in spatial memory. It could also regulate APP processing, activate PKC and MAPK. Inhibition of neuronal death markers, prevention of the fall in mitochondrial membrane potential and upregulation of neurotrophic factors and antioxidative activity rationalize it as an anti-AD agent [27]. Still other compounds that inhibit MAO are also under investigation for AD prevention [28].

## Treatments based on tau pathology

Tau is a microtubule-associated protein normally present in neurons. In AD, hyperphosphorylated tau forms the paired helical filaments (PHF). This process severely impairs axonal transport. A number of tau-focused targets for treatment of tauopathies such as AD, are emerging following the recent development of transgenic animal models expressing tau abnormalities and hyperphosphorylation.

### Prevention of phosphorylation of tau

Tau phosphorylation increases dramatically in AD, suggesting tau kinase inhibitors could be used as an anti-AD treatment. Phosphorylation of tau is controlled by different kinases and phosphatases. The activity of protein phosphatase (PP)-2A may increase dephosphorylation of tau. PP-2A also inhibits kinases such as MAPK, which phosphorylate tau. Cyclin-dependent kinase-5 (CDK5) is a kinase suggested to phosphorylate tau in AD. Transgenic mice in which CDK5 activity is activated (by overexpression of the p25 activator) in adult brain show evidence of a striking neurodegeneration with some tau pathology. It has been reported that the concentration of p25 is elevated in the human AD brain. Therefore, inhibition of CDK5 may suppress tau phosphorylation and prevent tangle formation. As report, inhibitors of CDK5 appear to have some influence on the development of pathology in some tau transgenic mice [29]. There are as yet no reports of the use of CDK5 inhibitors in humans.

Glycogen synthase kinase (GSK)-3β has also been suggested as a drug target to inhibit tangle formation. It is well established that this kinase can phosphorylate tau in cells in culture and in the brains of transgenic mice. This kinase is blocked by lithium, which has a long record as a mood stabiliser or for augmenting antidepressive therapy; it inhibits tau phosphorylation with beneficial effects in animal models [30]. It has been reported that lithium could be used for AD prevention, particularly in individuals at risk of presenile FAD, which has early onset [31]. The M1 muscarinic agonist AF267B (also referred to as NGX267) has been shown to inhibit GSK-3β activity and reduce tau pathology in transgenic mice. Two additional inhibitors of tau hyperphosphorylation that have shown modest effect in transgenic mouse models are propentofylline (PPF) and SRN-003-556. Although the exact mechanism of PPF is unknown, it reduced the active form of GSK-3β and prevented the hyperphosphorylation of tau. SRN-003-556 was able to reduce soluble tau that was hyperphosphorylated; however, no change was evidenced in existing neurofibrillary tangles [32].

Finally, activated MAPK has been reported to be associated with neurofibrillary tangles (NFTs) in human AD [33]. A rather nonspecific inhibitor of this kinase was used in tau transgenic mice, with apparently some beneficial results [33]. But as with GSK3 and CDK5, there are concerns that the multifaceted role of this kinase in cellular metabolism would appear to lower the probability that such inhibitors will make it into human studies.

### Prevention of the aggregation of tau

Tau is ordinarily a soluble protein, but forms insoluble, filamentous aggregates in the course of NFTs formation. The mechanisms responsible for the conversion of a normally soluble monomeric protein into the insoluble filamentous aggregates have been the subject of intense study and the target for some drug development. Other approaches to tau include blocking its aggregation, either directly or by inhibiting its proteolysis. Inhibitors of tau aggregation independent of phosphorylation have been found and tested in cell cultures [34]. Recent studies using cell models have demonstrated that certain drug inhibitors are able to prevent tau protein aggregation and even dissolve the developed aggregates, which include phenothiazines, anthraquinones, polyphenols, thiacarbocyanine dyes, N-phenylamines, thiazolyl-hydrazides, rhodanines, quinoxalines, aminothienopyridazines and so on [35]. Although these initial findings are promising, studies in vivo are still needed to demonstrate efficacy and safety of tau aggregate inhibitors.

### Prevent the misfolding of tau

In addition to tau aggregation, the misfolding of hyperphosphorylated tau proteins has also been suggested to contribute to the pathology of AD. Certain proteins are able to regulate and prevent improper folding of tau in order to avoid aggregation. The results from a study by Dou and colleagues [36] suggested that increasing the activation of molecular chaperones might prevent the misfolding of tau, which would then reduce the development of NFTs. Heat shock proteins have been shown to activate chaperones that prevent misfolding and even promote tau binding with microtubules [37]. Additional research is required to determine whether targeting tau chaperones would be able to produce significant benefit in humans.

### Tau immunotherapy

Over the past several years, there has been a growing interest in immunotherapies targeted at reducing tau levels as a strategy for treating AD. One of the possible immunotherapeutic approaches being considered is the selective reduction of pathological forms of tau. Asuni and colleagues demonstrated that immunization of mice expressing P301L-tau (JNPL3 mice) with a small phospho-tau peptide (amino acids 379–408 with S396/S404 phosphorylated) resulted in the production of antibodies that entered the brain, which reduced the extent and slowed the progression of the behavioural phenotype [38]. This group recently extended these studies by creating a new mouse model with accelerated development of tau pathology and immunizing with the same phospho-tau peptide. Using this model they demonstrated that immunization with this tau peptide resulted in a reduction in soluble and insoluble tau phosphorylated at S396/S404 and a significant attenuation of cognitive impairment [39]. Kayed R reported similar results when phosphorylated tau antigens were used with Freund’s-adjuvant (CFA) or with pertussis-toxin (PT) and found 40% reduction in NFTs, reversal in neurological deficits and 20% increase in microglia in mice [40]. A study from another group also demonstrated that immunization of other tauopathy mouse models with phospho-tau peptides also reduced tau pathology without significant side effects [41]. Given this finding, it would seem that a passive immunization protocol may be a better therapeutic approach.

## N-methyl-D-aspartate receptor (NMDA) antagonist

Glutamate is found in the neural pathways associated with learning and memory. Abnormal levels of glutamate may be responsible for neuronal cell dysfunction and the eventual cell death and subsequent cognitive impairment observed in AD. Age-related changes in NMDA receptors have been found in cortical areas and in the hippocampus in many species. In 2003, the FDA approved memantine (Namenda; Forest Laboratories, New York, NY), a low to moderate-affinity noncompetitive NMDA receptor antagonist, for the treatment of moderate to severe AD. Memantine appears to restore the function of damaged nerve cells and reduce abnormal excitatory signals by the modulation of the NMDA receptor activity. It is thought to block selectively the effects associated with abnormal transmission of the neurotransmitter glutamate, while allowing for the physiological transmission associated with normal cell functioning [42]. In patients with moderate to severe AD receiving a stable dose of donepezil, the addition of memantine results in significant improvement in cognitive, functional, and global outcomes compared with placebo [43]. Benefit was also shown with memantine monotherapy in an outpatient study. However, in comparison, greater cognitive and functional improvement was seen in recipients of dual therapy.

## Non-steroidal anti-inflammatory drugs (NSAIDs)

Microglial cells, closely related to the macrophage series of cells in the periphery, increase in size and number in the brain with AD. From this observation and the presence of complement in amyloid plaques, the concept of AD as an inflammatory disease has emerged. More than 20 epidemiological studies, some with a follow-up design and a good estimation of NSAIDs use via prescription data from pharmacies have suggested that the prolonged intake of NSAIDs may be associated with a reduced incidence of AD [44]. In a Canadian 10-year population-based cohort study, with the incidence of CIND (mild cognitive impairment or cognitive impairment, not dementia), AD, and all-cause dementia, it was shown that there is an association between NSAID use and a lower incidence of AD [45]. However clinical trials with potent NSAIDs (indometacin) led to high withdrawal rates as a result of gastrointestinal toxicity. Some trials with COX-2 selective (celecoxib and rofecoxib) or unselective (naproxen) NSAIDs or other anti-inflammatory drugs such as dapsone, hydroxychloroquine and prednisone have not shown a beneficial effect. Further trials with NSAIDs shown that NSAIDs have an adverse effect in later stages of AD pathogenesis, whereas asymptomatic individuals treated with conventional NSAIDs such as naproxen experience reduced AD incidence, but only after 2 to 3 years. Thus, treatment effects differ at various stages of disease.

## Estrogens

Estrogens are neuroprotective against oxidative stress, excitatory neurotoxicity, and ischemia in the brain. Studis shown that estradiol administration significantly ameliorates the neurodegeneration characteristic of AD in experimental rat model. This may be attributed to its powerful antioxidant, antiapoptotic, neurotrophic as well as its antiamyloidogenic activities. Merlo et al. reported that estrogen can activate matrix metalloproteinases-2 and −9 to increase beta amyloid degradation [46]. Receptors within the forebrain are found on the same neurons as cholinergic receptors specifically affected by the pathologic changes in AD. It is speculated that the withdrawal of estrogen in postmenopausal women results in a predisposition to AD. The use of estrogen during hormone-replacement therapy (HRT) has also been posited as a neuroprotective treatment for AD. Several small short-term randomized clinical trials and some epidemiologic studies [47] have suggested efficacy of hormone therapy in the treatment of mild to moderate AD. Results of a randomized controlled trial short-term hormone therapy with transdermal estradiol improved cognition for postmenopausal women with AD [48]. However, a study by Shumaker and colleagues indicated that postmenopausal women treated with estrogen plus progestin had an increased risk for dementia [49]. Thus, there is insufficient evidence to support use of HRT for prevention of AD and studies in AD with oestrogens (e.g.premarin, raloxifen) are now mostly in phase II.

## Nicotine

Nicotine is a cholinergic agonist that acts both post-synaptically and pre-synaptically to release acetylcholine, which is an alkaloid derived from the leaves of tobacco plants (Nicotiana tabacum and Nicotiana rustica). Nicotinic receptor densities are further attenuated in age-associated neurodegenerative disorders in the elderly, such as AD [50]. Numerous investigations, both in vivo and in vitro, indicate that nicotine can enhance neurone survival in response to a range of neurotoxic insults. Recent report that nicotine evoked improvement in learning and memory is mediated through neuropeptide Y Y1 receptors in rat model of AD [51]. Published studies in humans have reported the effects of intravenous or subcutaneous nicotine administration on people with AD. Significant improvements were reported in several cognitive tasks such as free recall, visual attention and perception and in mood although not on memory. There is also the possibility that nicotine might have a preventive action on AD, delaying the onset of clinical dementia by reducing the rate of neuronal loss or mitigating its functional consequences. There is also evidence that chronic nicotine in vivo and tobacco use is capable in reducing brain Aβ in elderly individuals although both the mechanisms of action remain uncertain [52]. These results suggest that central nicotinic cholinergic stimulation deserves further investigation as a possible treatment for AD. On the other hand, because nicotine has been related with adverse effects, especially concerning cardiovascular risks in elderly people, and also on sleep and behaviour it is important to further study the efficacy and safety of nicotine in patients with AD.

## Melatonin

Melatonin (N-acetyl-5-methoxytryptamine) is a tryptophan metabolite, synthesized mainly by the pineal gland. Melatonin has a number of physiological functions, including regulating circadian rhythms, clearing free radicals, improving immunity, and generally inhibiting the oxidation of biomolecules. Melatonin receptors appear to be important in mechanisms of learning and memory in mice, and it can alter electrophysiological processes associated with memory, such as LTP. It is generally accepted that melatonin deficit is closely related to aging and age-related diseases. Decreased levels of melatonin in serum and in CSF and the loss of melatonin diurnal rhythm are observed in patients with AD [53]. The first published evidence that melatonin may be useful in AD was the demonstration that this neurohormone prevents neuronal death caused by exposure to the amyloid beta protein [54]. Melatonin also inhibits the aggregation of the amyloid beta protein into neurotoxic microaggregates. Recent report has indicated that melatonin can alter lipid levels of mitochondrial membranes induced by amyloid beta protein [55]. Melatonin has been shown to prevent the hyperphosphorylation of the tau protein in rats. Studies in rats suggest that melatonin may be effective for treating AD [56]. In AD patients, melatonin supplementation has been suggested to improve circadian rhythmicity, and to produce beneficial effects on memory. Extensive clinical trials and studies with transgenic models are necessary to confirm the role of melatonin at the late pathological stage of AD.

## Cell transplantation and gene therapy

The degeneration of the cholinergic neurons in the nucleus basalis of Meynert leads to a reduction in the cholinergic innervation in the cortical and subcortical regions. This reduced neurotransmitter transduction correlates with the clinical and pathological severity of AD and is also a target for treatment. In AD rat model, transplantation of cholinergic-rich tissue or peripheral cholinergic neurons ameliorates abnormal behavior and cognitive function. But no clinical trials in AD patients have been initiated with this method. Lack of endogenous nerve growth factor (NGF) can lead to memory deficits, whereas NGF administration rescues neurons from injury-induced cell damage and leads to associated memory improvements [57] and thus NGF is good for gene therapy. In a phase I trial of ex vivo NGF gene delivery in eight individuals with mild AD [58], fibroblasts genetically modified to express human NGF were transplanted into the forebrain. After mean follow-up of 22 months in six subjects, no long-term adverse effects of NGF occurred. PET studies showed a widespread increase in glucose uptake by cortical neurons after 6~8 months. The cognitive decline was improved as evidenced by the mini-mental status examination (MMSE) and AD Assessment scale. Brain autopsy from one subject suggested robust growth responses to NGF. Although this is a small group and an open-label study with no placebo control, these results are very encouraging. And additional clinical trials of NGF for AD are warranted. Besides NGF, another candidate for gene therapy is Aβ-degrading enzymes and the animal experiments are also positive and the clinical trials are not being initiated yet.

## Other pharmacological therapies in clinical AD trails

### docosa-hexaenoic acid (DHA)

Epidemiological studies suggest that increased intake of the omega-3(n-3) polyunsaturated fatty acid DHA is associated with a reduced risk for AD [59]. DHA is the most abundant omega 3 fatty acid in the brain. Data from animal models support the hypothesis that DHA maybe an effective treatment for AD by means of anti-amyloid, antioxidant, and neuroprotective mechanisms [60]. Administration of omega-3 fatty acid for 12 months in a RCT with 204 patients with mild to moderate AD showed no delay in the rate of cognitive decline according to the MMSE or the cognitive portion of the AD Assessment scale. However, positive effects were observed in a small group of patients with very mild AD [61].

### Clioquinol

Metal chelation using clioquinol has been reported in a pilot study with 36 patients with AD to reduce the rate of cognitive loss in a double-blind, placebo-controlled, phase 2 clinical trial [62]. Clioquinol’s effect in this preliminary study is due to its ability to chelate zinc and copper associated with amyloid plaques. The mobilization and removal of brain amyloid is believed to be basis of its therapeutic effect. It was reported that clioquinol can reduce zinc accumulation in neuritic plaques and inhibit the amyloidogenic pathway in APP/PS1 transgenic mouse brain [63].

### Resveratrol

Resveratrol, a red wine polyphenol, is known to protect against cardiovascular diseases and cancers, as well as to promote anti-aging effects in numerous organisms. Some recent studies on red wine bioactive compounds suggest that resveratrol modulates multiple mechanisms of AD pathology. It has been recently suggested that resveratrol can be effective in slowing down AD development. As reported in many biochemical studies, resveratrol seems to exert its neuroprotective role through inhibition of Aβ aggregation, by scavenging oxidants and exerting anti-inflammatory activities [64]. In another paper, it demonstrates that resveratrol is cytoprotective in human neuroblastoma cells exposed to Aβ and or to Aβ-metal complex mainly via its scavenging properties [65]. Resveratrol appears to mimic the effects of calorie restriction (CR) or dietary restriction and to trigger sirtuin proteins [66]. Cardioprotection, chemoprotection against cancers, and anti-aging benefits all seem to be mediated through resveratrol and CR-induced activation of sirtuin and other proteins. Protection against Aβ toxicity in AD brains is also modulated by sirtuins. The efficacy of resveratrol in treating AD pathology depends on the extent to which resveratrol metabolites become bioavailable and influence both sirtuin-dependent and -independent signaling pathways in humans, and also depends on the next generation of clinical testing and research that will need to study the effects of resveratrol on a large number of human subjects.

## Conclusions

The pathogenesis of AD is a complex process involving both genetic and environmental factors; therefore development of effective disease-modifying drugs is proving to be a difficult task. Current therapies for patients with AD may ease symptoms by providing temporary improvement and reducing the rate of cognitive decline. Given the wide array of available molecular targets and the rapid progress toward identifying potential therapeutic compounds, the development of interventions that substantially delay the onset or modify the progression of AD can be anticipated.

## Abbreviations

Aβ: Beta amyloid; ACE: Angiotensin-converting enzyme; Ach: Acetylcholine; AD: Alzheimer’s disease; APP: Amyloid precursor protein; CDK5: Cyclin-dependent kinase-5; CSF: Cerebralspinal fluid; DHA: Docosa-hexaenoic acid; ECE: Endothelin converting enzyme; GSK: Glycogen synthase kinase; HRT: Hormone-replacement therapy; IDE: Insulin-degrading enzyme; LRP-1: Low-density lipoprotein receptor-related protein-1; LTP: Long term potentiation; MAO: Monoamine oxidase; MAPK: Mitogen activated protein kinase; NEP: Neprilysin; NFTs: Neurofibrillary tangles; NGF: nerve growth factor; NMDA: N-methyl-D-aspartate; NSAIDs: Non-steroidal anti-inflammatory drugs; PHF: Paired helical filaments; PKC: Protein kianse C; RAGE: Receptor for advanced glycation end products.

## Competing interests

The authors declare that they have no competing interests.

## Authors' contributions

H-Q Yang and Z-K Sun made equal contributions to conception and design, acquisition of data, and in drafting the manuscript. S-D Chen was the general supervision of the research group, acquisition of funding, and involved in revising it critically for important intellectual content. All authors read and approved the final manuscript.
